# Characterization of Interstitial Cajal Progenitors Cells and Their Changes in Hirschsprung’s Disease

**DOI:** 10.1371/journal.pone.0086100

**Published:** 2014-01-24

**Authors:** Zhi-Hua Chen, Yong-Chang Zhang, Wei-Fang Jiang, Cissy Yang, Gang-Ming Zou, Yu Kong, Wei Cai

**Affiliations:** 1 Shanghai Institute for Pediatric Research, Xin Hua Hospital, Shanghai Jiao Tong University School of Medicine, Shanghai, P. R. China; 2 The 32 Ward of Oncology, Hunan Provincial Tumor Hospital, the Affiliated Tumor Hospital of Xiang Ya Medical School of Central University, Changsha, P. R. China; 3 Institute of Neuroscience, Shanghai Institutes for Biological Sciences, Chinese Academy of Sciences, Shanghai, P. R. China; 4 Stanford University School of Medicine, Stanford, California, United States of America; MOE Key Laboratory of Environment and Health, School of Public Health, Tongji Medical College, Huazhong University of Science and Technology, China

## Abstract

Interstitial cells of Cajal (ICC) are critical to gastrointestinal motility. The phenotypes of ICC progenitors have been observed in the mouse gut, but whether they exist in the human colon and what abnormal changes in their quantity and ultrastructure are present in Hirschsprung’s disease (HSCR) colon remains uncertain. In this study, we collected the surgical resection of colons, both proximal and narrow segments, from HSCR patients and normal controls. First, we identified the progenitor of ICC in normal adult colon using immunofluorescent localization techniques with laser confocal microscopy. Next, the progenitors were sorted to observe their morphology. We further applied flow cytometry to examine the content of ICC progenitors in these fresh samples. The ultrastructural changes in the narrow and proximal parts of the HSCR colon were observed using transmission electron microscopy (TEM) and were compared with the normal adult colon. The presumed early progenitor (c-Kit^low^CD34^+^Igf1r^+^) and committed progenitor (c-Kit^+^CD34^+^Igf1r^+^) of ICC exist in adult normal colon as well as in the narrow and proximal parts of the HSCR colon. However, the proportions of mature, early and committed progenitors of ICC were dramatically reduced in the narrow segment of the HSCR colon. The proportions of mature and committed progenitors of ICC in the proximal segment of the HSCR colon were lower than in the adult normal colon. Ultrastructurally, ICC, enteric nerves, and smooth muscle in the narrow segment of the HSCR colon showed severe injury, including swollen vacuola or ted mitochondria, disappearance of mitochondrial cristae, dilated rough endoplasmic reticulum, vesiculation and degranulation, and disappearance of the *caveolae* on the ICC membrane surface. The contents of ICC and its progenitors in the narrow part of the HSCR colon were significantly decreased than those of adult colon, which may be associated with HSCR pathogenesis.

## Introduction

Interstitial cells of Cajal (ICC) located between gut nerve fibers and smooth muscle cells play a critical role in gastrointestinal motility. They mediate both excitatory and inhibitory neuromuscular neurotransmission [1], [2]. Many studies support that ICC have regenerative capacity, which could restore their networks after gut injury [3], inflammation [4], [5] surgical transection and anastomosis [6], [7]. ICC depletion is probably a key point in the pathogenesis of these disorders, and patients would benefit from its reversal. However, in other gastrointestinal motility disorders, the number of ICC is extremely reduced and difficult to reverse. These disorders include achalasia, diabetic and idiopathic gastroparesis, mechanical ileus, and intestinal pseudo-obstructions. Depletion of ICC could result from severe injury of mature cells and/or impaired regeneration of progenitor cells.

Hirschsprung’s disease (HSCR) is a congenital disorder of the colon, causing chronic constipation. Its incidence is about 1∶5000 in the United States, but around the world incidence ranges from about 1∶1000 to 1∶10,000. Children with HSCR often have the following symptoms: delayed passage of meconium (>24 hours from birth), neonatal bowel obstruction (abdominal distension, green or yellow vomiting), constipation that does not respond to oral medicines, poor growth, and sometimes loose bowel movements with blood and accompanying fever. Currently, clinical diagnosis of HSCR is based on the lack of ganglion cells at the myenteric and submucosal plexus. Previous reports have shown that there were alterations of ganglion cell genes, such as RET, PHOX2B, HOX and NGR3 which are associated with the Hirschsprung’s disease [7]–[10]. However, these results are obtained from blood drawn from patient and not fresh colon tissue. Researchers have tried the therapy of enteric nervous system stem cells (ENSSCs), but previous animal models have failed to completely restore the intestinal physiological function after transplantation [11]–[14]. We suggest that there is another critical point in HSCR guts, the ICC network that requires repair. The number of ICC has been found to be remarkably reduced in the narrow segment of the HSCR colon, which is pathologic [15]–[18].

ICC develop prenatally from c-Kit^+^ (also named CD117) mesoderm mesenchymal progenitors [19], [20]. Intramuscular ICC of the foregut may also be derived from ventrally emigrating neural tube cells [21], [22]. Progenitors of ICC committed to become mature ICC have been reported during the early postnatal period [23], [24]. ICC from mouse gut showed proliferation that was SCF and IGF-I dose-dependent and time-limited [25], [26]. Recently, two studies brought to light that defects of ICC can be repaired by bone marrow mesenchymal stem cells (BMSC) [27], [28]. It was speculated that ICC progenitors may persist in adulthood although their numbers are likely very small. Their malignancy can lead to gastrointestinal stromal tumors (GIST). GIST may be due to activating gene mutations in c-Kit [29], [30] and originate from c-kit^low^ ICC progenitors [31]. Additionally, a CD34^+^ subset of ICC may give rise to GIST [32]. The only clinically effective treatment for c-Kit^+^CD34^+^ GIST other than surgical resection is Imatinib, which is a tyrosine-kinase inhibitor. Lőrincz *et al* first reported in mouse gut that the phenotype of early progenitors of ICC was c-Kit^low^CD44^+^CD34^+^Insr^+^Igf1r^+^, and the phenotype of committed progenitors of ICC was c-Kit^+^CD44^+^CD34^+^Insr^+^Igf1r^+^ [32]. In another report, it was found that clonogenic c-Kit^low^CD44^+^CD34^+^ cells from mutant mice were capable of self-renewal and differentiation into mature ICC (c-Kit^+^CD44^+^CD34^−^Insr^−^Igf1r^−^) in special medium for stem cells [33]. From early committed progenitors of ICC to mature ICC, the phenotype change seems to reflect the maturing process of ICC [34].

We address the following questions in this study: whether the two kinds of progenitor phenotypes of ICC in mouse gut also exist in human gut with aging; what abnormal changes of ICC progenitors including their quantity and ultrastructure present in HSCR colon. To learn the biological changes in HSCR lesion colon, we collected fresh surgical resection samples including adult normal colon as well as narrow and proximal segments of congenital HSCR colon. We identified the progenitor of ICC using laser confocal microscopy, and further applied flow cytometry to examine the content of ICC progenitors in different fresh samples. The progenitors were sorted from normal colon to observe morphology. We found that ICC progenitors exist in the colon of humans ranging from children to the elderly, but the proportions of mature and progenitors of ICC were significantly reduced in the narrow part of the HSCR colon. TEM showed that in the narrow part of the HSCR colon the ultrastructure of ICC was subjected to severe injury, and the enteric nervous and smooth muscle cells were not spared.

## Materials and Methods

### Human Samples Collection

This protocol was approved by the Institutional Review Board of Xin Hua Hospital affiliated to Shanghai Jiao Tong University School of Medicine, in accordance to generally accepted international practice. Written informed consent was obtained from all patients or their parents. The inclusion criteria are: 1) classic syndromes of HSCR in neonatal period; 2) common type HSCR cases; 3) cases confirmed by full-thickness biopsy before radical surgery; 4) underwent one-Stage transanal Soave pullthrough surgery; 5) improvement of life quality after surgery: normal feeding, abdominal distension disappeared, self-defecation, growth and development of children up to the normal level of their peers. The exclusion criteria are: 1) absence of parental consent; 2) neuronal intestinal dysplasia; 3) abnormal bowel function after surgery such as recurrence of constipation or/and bloating; 4) loss of follow-up. Based on above criteria, we began to collect the samples from November 2011 to June 2012 in Xin Hua hospital. After the patients discharged from hospital, they performed follow-up about their bowel function at out-patient department every 3 months.

Common type accounts for about 75% of HSCR pathological types, the aganglionosis region extending from the anus to the sigmoid of colon. The short type (S-type) accounts for about 8% of HSCR pathological types and the aganglionosis region includes the part of the internal sphincter. Total colonic aganglionosis (TCA) and long-segment aganglionosis (LSA) may be more serious in pathological states. Neuronal intestinal dysplasia (NID) is another special type of HSCR. Therefore, they were not included in this study. For the same baseline comparison, we only chose the common type and their lesions were at the rectum and sigmoid colon. Samples of the proximal segments were collected from the distal descending colon. This indicates the tissue samples of all cases were collected from similar locations. These common type HSCR cases had typical clinical symptoms. Before surgery, diagnostic criteria by biopsy for the narrow segment (pathologic stenosis part) required a lack of ganglion cells in Submucosal Meissner’s plexus (MP) and Myenteric Auerbach’s plexus (AP) accompanied by nerve fiber proliferation, thickening and derangement. At the sphincter level of the anal canal, there usually exists very few or no ganglion cells. Therefore, samples of the narrow segment were collected from a location 3 cm above the dentate line. We collected the samples of narrow and proximal segments from each HSCR patient simultaneously. The proximal part was collected from the surgical reconstruction site and diagnosed as well-developed ganglion by frozen section in operation. Children with HSCR gradually returned to normal bowel function after surgery, if serious constipation reoccurred, the participant was removed from the group. Based on having normal bowel function, we considered the tissue from this position to be relatively normal.

As different age group, 11 adult normal colons were collected. The adult patients had left colon cancer underwent radical surgery of removing not only the colon cancer but also much of the normal colon. Before operation, physical exam showed that all adult patients had normal glycemia and bowel function, which was confirmed by anorectal manometry. The samples were collected from a position distant to the cancer region and examined in surgery pathology assays as normal colon tissue. Sample collection from resections did not affect patient care. All of samples were used for immunofluorescence, flow cytometry, culture *in vivo* and TEM (Flowchart S1).

### Whole-mount Frozen Section for Laser Confocal and 3D Reconstruction in-situ

Whole-mounts of the colon tissue of HSCR (n = 5) and adult normal colon samples (n = 5) were fixed with O.C.T. compound (SAKURA Tissue - Tek, 4583) as soon as the specimens were excised and frozen at −20°C overnight. Frozen tissues were cut into 35 µm slices with the cryostat and mounted on coated slides. They were fixed in 4% paraformaldehyde for 10 minutes. After blocking with in 5% albumin bovine serum (BSA) for one hour, colon samples were incubated with the primary antibodies overnight in a wet-box at room temperature. The primary antibodies were rabbit polyclonal anti-c-Kit (Abcam, 1∶200), goat polyclonal anti-CD34 (Santa Cruz, 1∶200), and mouse monoclonal anti-Igf1r (Abcam, 1∶200). Secondary antibodies were donkey anti-rabbit IgG conjugated CF488 (Biotium, 1∶200) to c-Kit, donkey anti-mouse IgG conjugated CF 633 (Biotium, 1∶200) to Igf1r, and bovine anti-goat IgG conjugated CF543 (Biotium, 1∶200), applied for 1 h at room temperature. Slides were washed with PBS three times and mounted using fluorescent mounting media for observation. All immuno-staining slides were examined, and images were taken by laser confocal microscopy using LSM 510 (Carl Zeiss Microimaging, Thornwood, NY). All sections were evaluated by two independent pathologists blinded to the disease outcome. Aim-image examiner by Zeiss LSM data server was used to carry out post-acquisition modification, limited to maximum-transparency projection, noise reduction, assignment of pseudocolor, and adjustment of brightness and contrast. 3D reconstruction pictures were made by Imais software.

### Single Cell Suspension Preparation

All 11 adult normal colon samples, 11 narrow and proximal segments of HSCR samples were used to prepare single cell suspension for flow cytometry detection and sorting, with the exception of samples used for detecting immunofluorescence and TEM. To minimize the effects of ischemia, as soon as the resection was removed from the body, about 3×3 cm of the colon muscularis was collected as quickly as possible in the operating room and immediately placed into ice-cold Medium 199 to be delivered to the lab. The mucosa, serosa layers, and hanging fat attached on the sample were cleaned away, and only the muscularis was used.

After rinsing with MEI (Multiple Electrolytes Injection, Baxter) for 5 minutes, the tunica muscularis was minced on ice to about 1×1 mm in size, washed with 45 ml MEI, centrifuged for 10 minutes at 1500 rpm (400×g) at 4°C, and the supernatant was aspirated. In this study, enzyme solution containing collagenase SERVA NB4 (17454.01), which contains class I and class II collagenases and a balanced ratio of proteolytic activities, requires calcium ions both for full catalytic activity and binding to the collagen molecule, prepared before each use. The precipitate was loaded into cap flasks with 15 ml Medium 199 (Sigma, St. Louis, MO) and SERVA NB4 1 mg/mL, incubated in shaker at 37°C for almost 1–1.5 hour. During this time the tissue fragments were gently dispersed and separated into single cells. After digestion, 35 ml MEI was added to the digested tissue. The sample was transferred to a centrifuge tube and centrifuged for 10 minutes at 1500 rpm at 4°C. The supernatant was then discarded, and the precipitate was suspended with Medium 199. The resuspension was filtered by filters (40 µm, BD Falcon) to remove undigested tissue crumbs, and the filtrate was centrifuged for 5 minutes at 300×g. The supernatant was aspirated completely. The precipitate was suspended with M199 again to create a single-cell suspension.

### Flow Cytometry

For removal of interference, macrophages and dendritic cells were labeled with superparamagnetic monoclonal antibodies against CD11b and CD11c (Miltenyi Biotec, Auburn, and CA), respectively. Those immune cells were depleted by immunomagnetic selection (Miltenyi Biotec). After the pre-treatment, the nucleated cells were suspended up to 10^6^ cells per 80 µl of buffer and 20 µl FcR blocking reagent was added to the cells, which were then incubated at room temperature. After 15 minutes, the conjugated antibodies were added to every 100 µl/test and incubated for 30 minutes at room temperature in the dark. These conjugated antibodies included 20 µl/test PE/Cy7-conjugated-mouse anti-human c-Kit (Biolegend), 10 µl/test PerCP-conjugated-mouse anti-human/mouse Igf1r (R&D system), 20 µl/test PE-conjugated-mouse anti-human CD34 (Beckman coulter). These three conjugated antibodies used in this study would never evoke the potential cross-talk between the fluorescence by illumination at 488 and 543 nm. Mast cells and other leukocytes were labeled with monoclonal FITC-anti-CD45 antibodies (eBioscience, San Diego, CA). The red blood cells mixed in whole sample were removed by 500 µl/test RBC lysis (OptiLyse C, Beckman Coulter) for 5–10 minutes on ice. After washing with 15 ml MEI, the cells were suspended in M199 with 2% FBS, filtered again by 40 um filters before flow cytometry analysis or sorting.

In order to obtain the most reliable data *in vivo*, all manipulation times including single cell suspension preparation and flow cytometry analysis or sorting were controlled within 5 hours after samples were apart from body, throughout the entire experiment.

### Flow Cytometry Parameters for Sorting and Assay

Quantification of the single-cell suspensions from colon muscularis samples expressing c-Kit, CD34 and Igf1r were analyzed and sorted by Moflo XDP (Beckman-Coulter) with a 100 µm orifice. It was equipped with a 488-nm Ar laser, a photodiode to measure light scattered at low forward angles (forward scatter), and photomultiplier tubes to measure orthogonally scattered light (side scatter), plus four wavelengths of fluorescence at 525 nm (used for the detection of antibodies tagged with FITC and AF488), 575 nm (used for PE), 675 nm (used for PerCP) and >750 nm (used for PE/Cy7). Proper operation of the instrument was verified before each experiment by analyzing standard reference beads. For final purification of ICC and their progenitors, the enriched cells were resorted using a one-drop sort mode. The sorted cells were identified by Moflo XDP using FCM mode. The flow cytometry data were analyzed with Summit5.3 (Beckman-Coulter).

For each experiment, at least 1**×**10^6^ cells were measured. To remove the effect of cell and tissue debris derived from the colon samples digestion process, we set a gate of R1 in flow cytometry. Cells inside of R1 are considered living cells. R2 is a gating scheme to exclude hematopoietic marker CD45 positive cells. A panel of 3 colored monoclonal antibodies (C-kit, CD34, Igf1r) served as isotype control for the identification of every marker that was used later in this study. Each specimen was considered positive or negative compared with the degree of the same specimen stained with the isotopic antibody as well as the blank control.

Refer to previous report (33), which identified c-Kit^low^ population as between c-Kit^+^ and c-Kit^−^ populations were detected by flow cytometry. In particular, on the abscissa of anti-c-Kit, the existence of a c-Kit^low^ population can clearly identified. We also used the total cell population expressing CD34^+^/Igf1r^+^ to distinguish the distribution of stained cells from nonspecific immunoreactivity.

### Laser Confocal Microscopy to Image Single ICC Progenitors in Total Cell Population

Before sorting, to obtain the merged image of ICC progenitors with three markers of fluorescence, 3×10^5^ single cells from normal adult colon muscularis were enriched onto glass slides by cytospin. After that, they were fixed in 4% paraformaldehyde for 10 minutes, conjugated with antibodies of c-Kit, CD34, Igf1r, incubated for 30 minutes at room temperature in the dark. Cell nuclei were stained with DAPI for 10 minutes, washed by PBS three times, and the slides were examined and imaged by laser confocal microscopy using LSM 510 (Carl Zeiss Microimaging, Thornwood, NY). Non-progenitors of ICC used as a negative control.

### Assessing the Accuracy and Cell Integrity after Sorting

After sorting, the cells were collected in MEI containing 2% FBS and thrown onto glass slides by cytospin. The slides were fixed in 4% paraformaldehyde for 10 minutes and cell nuclei were stained with DAPI for 10 minutes. Slides were washed with PBS, and cell fluorescent morphology was observed by LSM 510 laser confocal microscopy (Carl Zeiss Microimaging, Thornwood, NY).

### Living Colon Muscularis Cell Culture

The cells of colon muscularis from adult normal colon (n = 3) or HSCR samples (n = 3) were cultured (6×10^5^ cells per well) in Medium 199 containing 10% FBS, penicillin (100 U/ml) and streptomycin (100 mg/ml), with or without supplement with human recombinant SCF (50 ng/ml, R&D system), human recombinant IGF-I (100 ng/ml, R&D system), at 37°C in a humidified atmosphere of 5% CO2 in air. The medium was replaced at 2-day intervals.

### Transmission Electron Microscopy

Random fresh tissue samples were collected from the adult normal colon group (n = 3) and the HSCR patient (narrow and proximal segment) group (n = 3). 2×1×1 mm samples were fixed in 2.5% glutaraldehyde in 0.1 M cacodylate buffer (pH 7.4) for 2 hours at room temperature. They were post-fixed in 1% OsO_4_ in 0.1 M cacodylate buffer and then dehydrated by ethanol through the following steps: 30% for 10 min, 50% for 10 min, 70% to overnight, 80% 20 min, 90% for 20 min, then the samples were washed three times in 100% ethanol at 20 min/each and two times in acetone at 30 min/each. Infiltration was done by the ratio of resin: acetone at 1∶1 for the first hour, ratio of resin: acetone at 2∶1 for the second hour, pure resin for another hour, and finally pure resin overnight. The colon samples were embedded in a 70°C oven overnight. The 70% ethanol was saturated with uranylacetate for contrast enhancement.

The specimens were embedded in EMBED-812 EMBEDDING KIT (Electron Microscopy Sciences). Ultrathin sections (70–80 nm) were cut on an Ultracut ultramicrotome (Leica UC6, Germany), mounted on pioloform-coated 50 mesh grids, and contrasted with lead citrate for 5 min. Ultrathin sections and replicas were observed and examined with an electron microscope (JEOL - 1230).

### Statistical Analysis

To quantify proportions of cells by flow cytometry, the number of events shown in R1 region in the flow cytometry assay was limited to 100,000. The values obtained by the events in R5, R6, R7 region were divided by the events in R1 region to represent the percent of mature, early, and committed progenitors of ICC in total live colon muscularis cells, respectively. These data are expressed as Mean ± SD, analyzed with SPSS11.5 (SPSS, Inc., Chicago, IL, USA). The data from the narrow and proximal segment of HSCR colon were analyzed as self-controls by paired-sample *t* tests. The data from the proximal segment of HSCR and adult normal colon as a different age control were ranked by independent-sample *t* tests. The data from male and female patients were analyzed by paired-sample *t* tests. A probability value of *P*<0.05 was used as a cut-off for statistical significance.

## Results

From November 2011 to June 2012, there were 44 HSCR patients admitted to our hospital, 36 cases of them met the inclusion criteria, but only 14 cases participated in this research with parents’ agreement. The 14 cases had follow-up in outpatient department every 3 months. Till October 2013, all of 14 cases had follow-up for more than one year and continued to do it. The longest follow-up time is 24 months and the shortest time is 16 months. During these time, two of them were excluded since sometimes recurrence of constipation or/and bloating when they got a cold, needed laxative treatment or anal discharge. One of them lost of follow-up. So only the 11 cases of HSCR patients were analyzed. These 11 cases performed same operation of one-Stage transanal Soave pullthrough surgery, after more than one year of growth and recovery they restored their self-defecation (“Bowel Function in Follow-up” Column in **Table S1 in File S1**).

Patients’ age of the HSCR group (6 male, 5 female) ranged from 3 to 36 months, and the average age was 10.36 months **(Table S1 in File S1)**. Patient age in the normal adult colon group (6 male, 5 female) ranged from 39 to 94 years, with an average age of 63.53 years **(Table S2 in File S1)**. The narrow segment was termed the lesion site of HSCR. About the surgical time in these 11 patients, three of them were 13 months, 21 months and 36 months, respectively **(Table S1 in File S1)**. Their symptoms of constipation and bloating before treatment were not very serious so they delayed to operate more than one year age. The other kids were all younger than 12 month (n = 8), their symptoms were more serious than the older before treatment so that they performed operation relatively early. We analyzed firstly the proportion difference of ICC and progenitors in proximal and narrow segment between younger and older 12 months **(Table S3 and S4 in File S1)**. Whatever in proximal or narrow segment, the result showed there was no difference between younger and older 12 months groups (*P* > 0.05). So the 11 cases were generalized in one group to analyze.

The proximal part was termed the site for surgical reconstruction with descending colon which was identified by well-developed ganglion by frozen section during the operation **(Fig. S1).** The diameter of the narrow part of the HSCR colon was approximately 1–1.5 cm, and the proximal part was 2–2.5 cm. The sample collection procedure and data comparison are shown in flow chart –1.

### Phenotype Analysis of Human ICC Progenitors by Laser Confocal Microscopy

**Figure 1:**
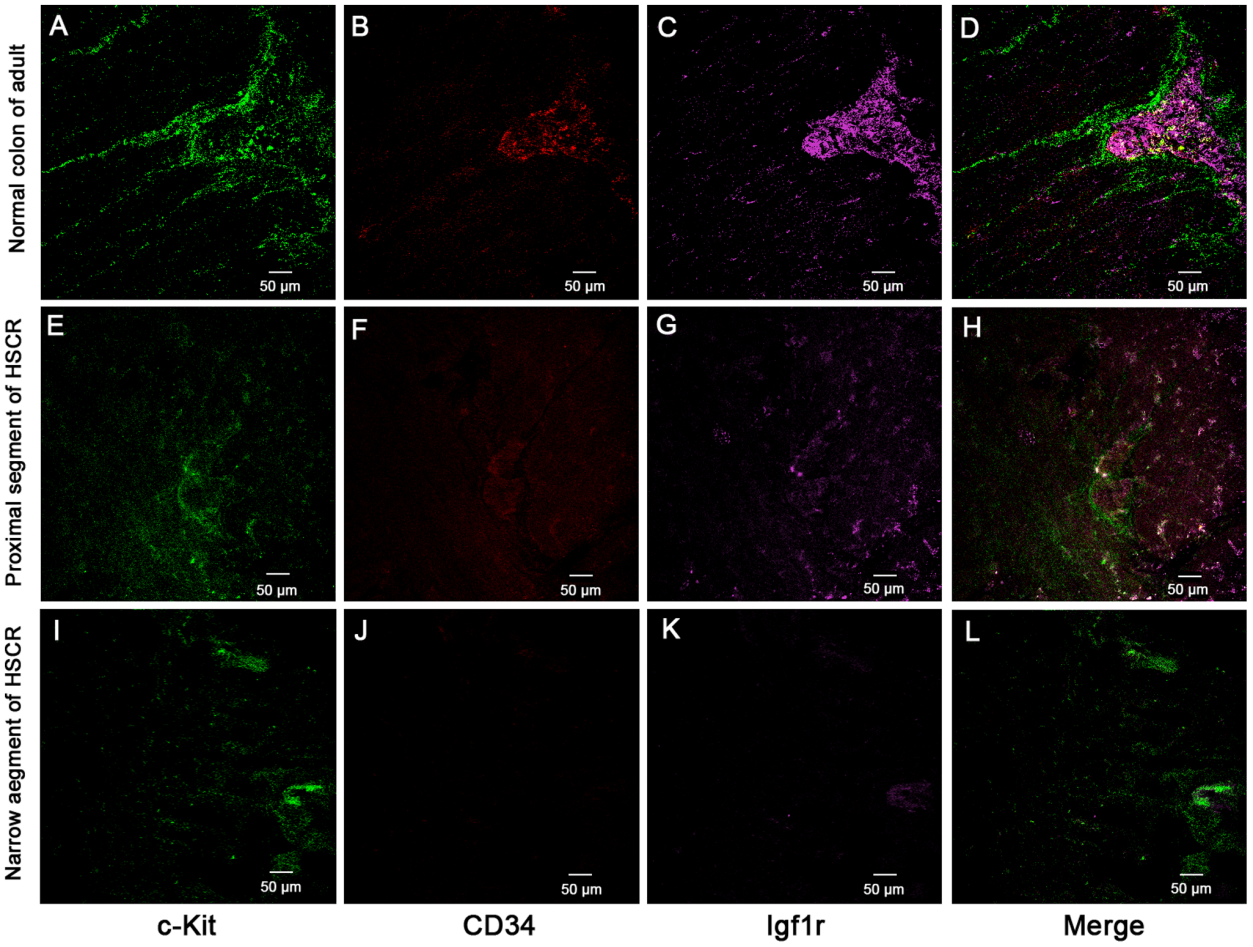
Mature and progenitor ICC located in human adult normal colon by laser confocal. (**A–D**) In AP of normal adult colon, the c-Kit positive mature ICCs connected to each other formed extending chords and around AP. ICC progenitors were co-localization of c-Kit^+^CD34^+^Igf1r^+^ found by overlapping fluorescence images. (**E–H**) In the proximal segment of HSCR, c-Kit positive cells and some of c-Kit^+^/CD34^+^/Igf1r^+^ cells were found occasionally. (**I–L**) In the narrow segment of the HSCR colon, it was very difficult to find positive ICC and their network structure was damaged, the c-Kit^+^/CD34^+^/Igf1r^+^ cells could not be located by overlapping fluorescence images. Green, red and pink fluorescence represent c-Kit, CD34 and Igf1r, respectively.

**Figure 2:**
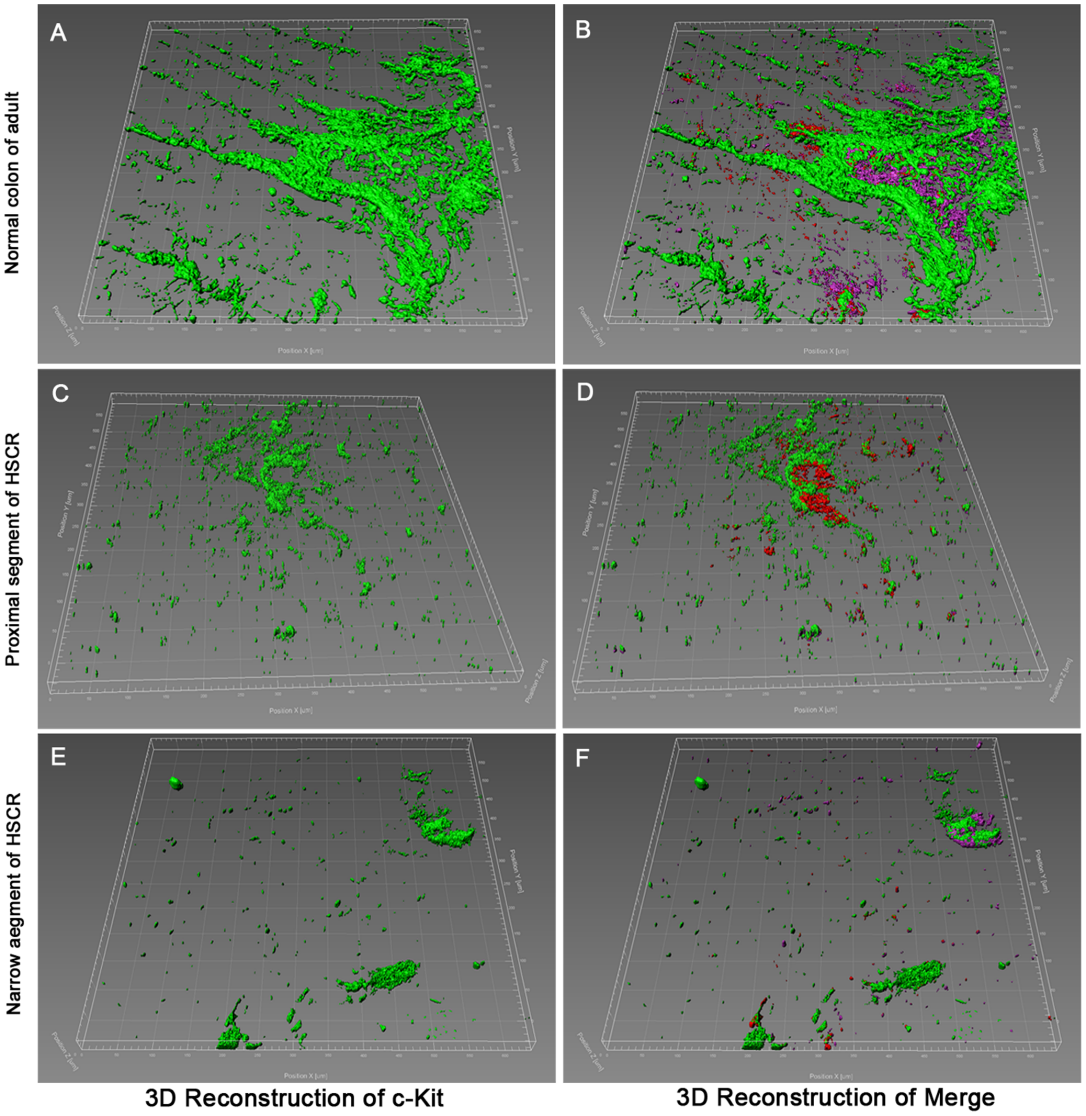
Immunofluorescent illustrations and 3D reconstruction in narrow and proximal segments of HSCR colon, compared with normal adult colon. (**A, B**) The 3D network structures of c-Kit^+^ and c-Kit^+^/CD34^+^/Igf1r^+^ cells were clearly visible in AP of normal adult colon. (**C, D**) The 3D structures in proximal segment of HSCR were not well formed. (**E, F**) The 3D structures in narrow segment of HSCR were completely destroyed.

In the whole-mount frozen sections of human adult normal colon, the c-Kit positive mature ICC were abundant and connected to each other formed extending chords **(** **Fig. 1A–D** **)** and 3D network structure **(** **Fig. 2A, B** **)**. In the merged image by laser confocal microscopy, at the Auerbach’s plexus (AP) level between circular and longitudinal smooth muscle layers, some c-Kit positive cells were found to be CD34^+^/Igf1r^+^ **(** **Fig. 1A–D** **)**. The enteric nerve also expressed Igf1r that displayed fluorescent in AP field. However, they didn’t expressed c-Kit and CD34. Clearly, the progenitors of ICC were located inside of the AP **(Fig. S2).** However, in the narrow segment of the HSCR colons, it was very difficult to detect c-Kit positive mature ICC and the c-Kit^+^/CD34^+^/Igf1r^+^ cells could not be located by overlapping fluorescence images in AP **(** **Fig. 1I–L** **)**. The 3D network structures of c-Kit positive cells were damaged in this segment (**Fig. 2E, F** **).** In the proximal segment, c-Kit positive mature ICC **(** **Fig. 1E** **)** and some of their 3D network structures of c-Kit were observed **(** **Fig. 2C, D** **).** In its AP level the c-Kit^+^/CD34^+^/Igf1r^+^ cells were found occasionally **(** **Fig. 1E–H** **)**.

### Flow Cytometry Analysis and Sorting of Human ICC Progenitors by Moflo XDP

**Figure 3:**
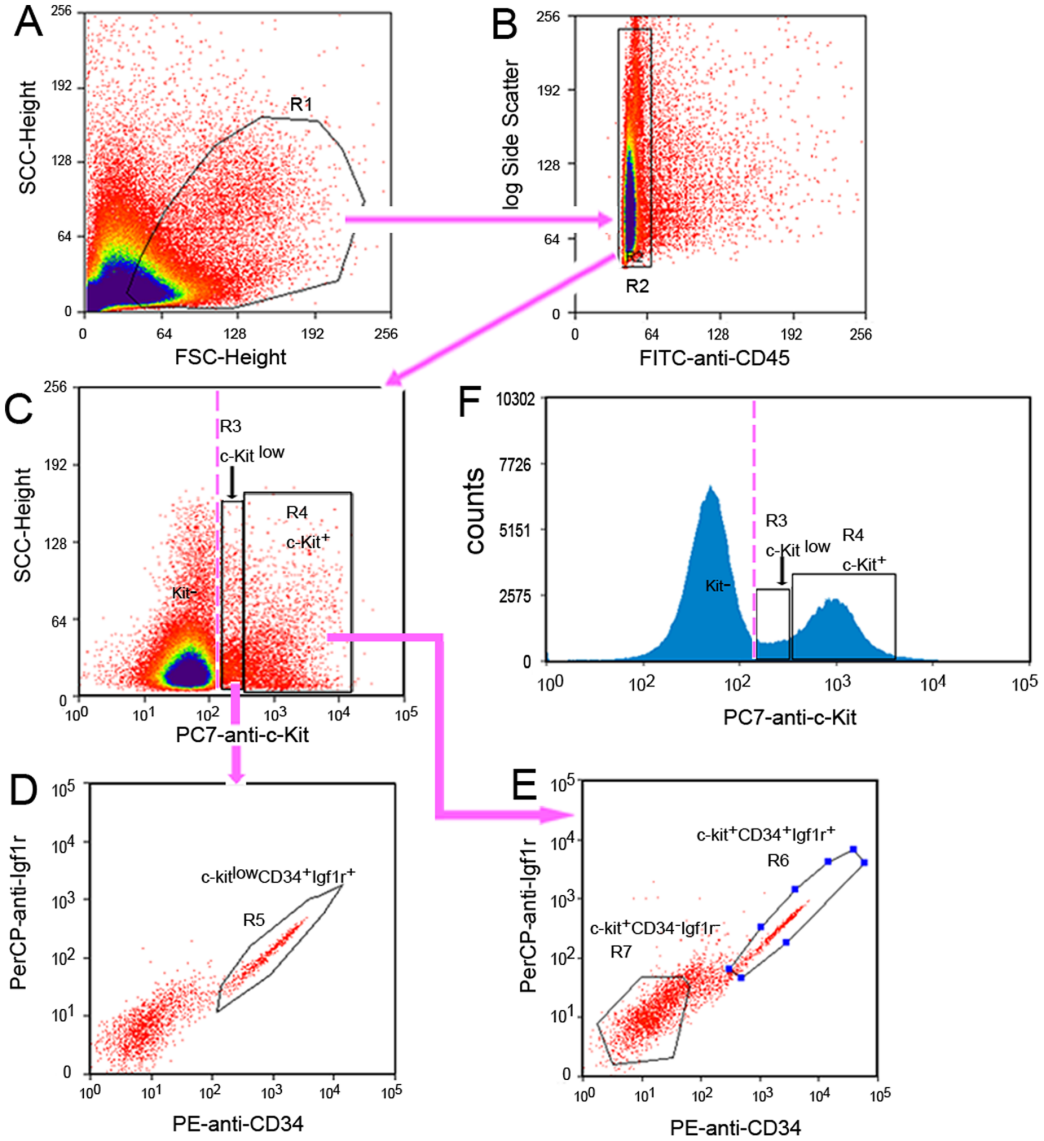
Flow cytometry sorting pathway to purify the mature, early and committed progenitors of ICC. Analysis following gating: (**A**) Selection of living mononuclear cells on the histogram with Side Scatter (SSC)/Forward Scatter (FSC). R1 gate was used to select the cells with light scatter properties characteristic of live cells. (**B**) R2 gate was used to select the cells not expressing macrophage markers (F4/80, CD11b) and the general hematopoietic marker CD45 (FITC- cells): gating on histogram SSC/CD45. (**C**) Detection of ICC phenotypes: the c-Kit^+^ cells population was gated in R4 and further analyzed in step **E**; the **c-**Kit^low^ cells population was gated in R3 and further analyzed in next step **D**. (**D**) The cells in R5 were **c-**Kit^low^CD34^+^Igf1r^+^. (**E**) The cells in R6 were **c-**Kit^+^CD34^+^Igf1r^+^, and the cells in R7 were c-Kit^+^CD34^−^Igf1r^−^. (**F**) To confirm the presence of the c-Kit^low^ population (R3) reliably between c-Kit^−^ and c-Kit^+^ populations (R4). Pink discontinuous straight line was used as the dividing line between c-Kit^−^ and c-Kit^low^ populations.

We identified human ICC progenitors by analyzing the expression of c-Kit, CD34, and Igf1r in enzymatically dispersed colon tunica muscularis by Moflo XDP **(** **Fig. 3** **)**. First, ICCs were identified as strongly c-Kit^+^ cells not expressing any of the myeloid markers F4/80, CD11b or the hematopoietic marker CD45 **(** **Fig. 3A–C** **)**. Between c-Kit^+^ (R4) and c-Kit^−^, there was a small but clearly recognizable population (R3) with low level of c-Kit defined as a c-Kit^low^ population **(** **Fig.** **3C, F** **)**. Upon further analysis, there was a fraction of c-Kit^low^ cells (R5) positive for CD34 and Igf1r **(** **Fig. 3D**), which were considered to be early progenitors of ICC. There was another fraction of c-Kit^+^ cells (R6) also positive for CD34 and Igf1r **(** **Fig. 3E** **)**, which were considered to be committed progenitors of ICC. The cells in R7 region in flow cytometry assay only expressed c-Kit^+^ but were negative for CD34 and Igf1r and were considered to be mature ICC.

The cells in R5 and R6 region in flow cytometry assay were the double-positive populations, but they occurred along the diagonal. To distinguish such distribution of stained cells from nonspecific immunoreactivity, we also analyzed the total cell population (R1) expressing CD34^+^/Igf1r^+^ to identify the location of nonspecific immunoreactivity **(Fig. S3)**. The location of double-positive CD34^+^/Igf1r^+^ cells was in R9 region in flow cytometry assay. The nonspecifically labeled cells located in R8 were very different from the population in R9 region in flow cytometry assay. We were able to detect the presence of mature, early and committed progenitors of ICC in both normal adult colon and HSCR colon samples, but the proportions of these cells differed between the groups.

### Characterization of Single Human ICC Progenitor Cells in Suspension

We successfully purified the ICC progenitor from fresh colon muscularis from clinical resection through cell sorter Moflo XDP cell sorter (Beckman-Coulter). Inevitably, some of cells had mechanical damage in sorting. The proportion of ICC progenitors is very lower in muscularis, so every time only 10^3^–10^4^ cells were harvested by sorting. The sorted cells were placed onto glass slides by cytospin, stained with DAPI and washed with PBS. This process would result in cell loss and damage. Therefore, the cell number harvested to observe was less than the number expected. Under the laser confocal, the cell distribution was very sparse. High magnification was used so that only one cell was shown under the microscope each time to better visualize the morphology.

**Figure 4:**
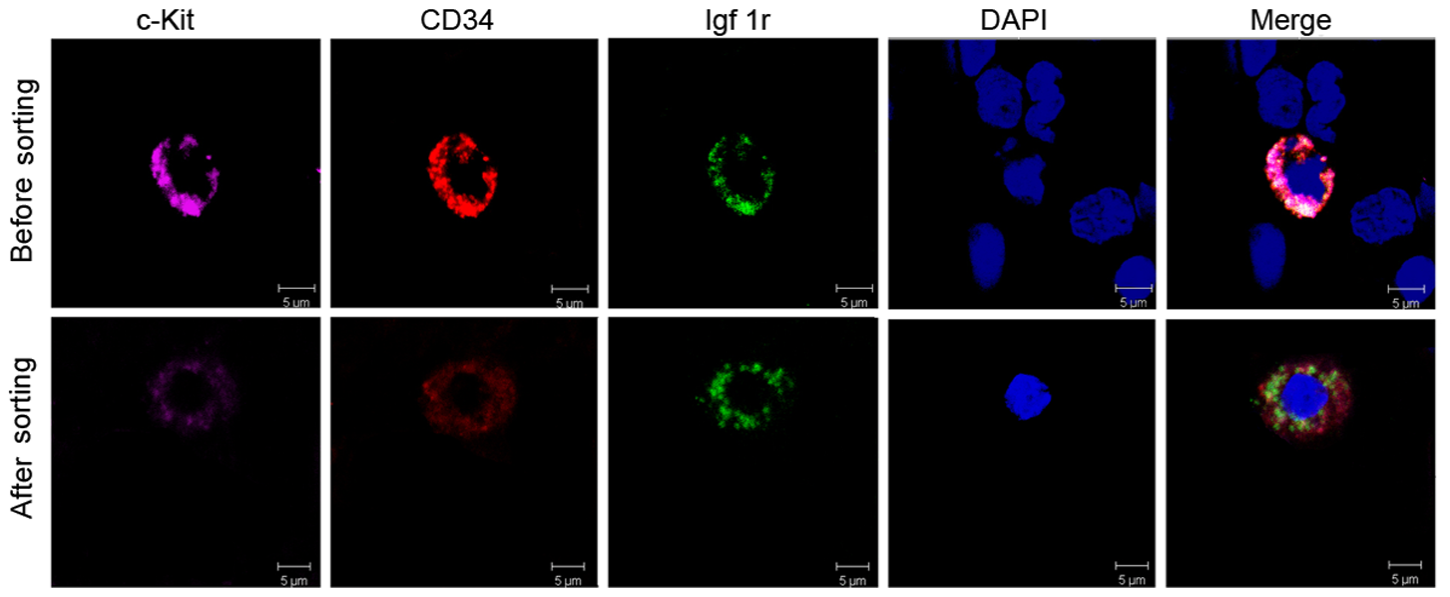
Morphology of single normal human ICC progenitor before and after sorting in suspension. Upper lane: before sorting, laser confocal microscope displayed phenotypes of ICC progenitor in total cell population from adult normal colon. ICC progenitors showed fluorescence of c-Kit (pink), CD34 (red) and Igf1r (green), merged with blue nuclear stained DAPI. At the same time, other cells only show DAPI staining as negative control, without other fluorescence. Lower lane: after sorting, laser confocal microscopy detected the single intact ICC progenitor with three-color fluorescence. But after immunofluorescence-activated sorting, the fluorescence of ICC progenitors recessed.

Before and after sorting by Moflo XDP, immunofluorescence examination by laser confocal microscopy revealed the morphological feature of integral human normal ICC progenitor with greater precision and detail **(** **Fig. 4** **)**. The cells are simultaneously positive for c-Kit, CD34 and Igf1r with relatively large nuclei. Before sorting, with the exception of ICC progenitors with immunofluorescence, the other cells without other fluorescence only showed DAPI staining as a negative control. The diameter of these cells measured by fluorescence imaging was about 12–15 µm. After immunofluorescence-activated sorting, characterization of the ICC progenitors was still possible, despite a notable reduction in fluorescence. The ICC progenitor from HD colon had same morphology on fluorescence, but it is not shown here.

### Proportions of ICC Progenitors are Severely Reduced in Hirschsprung’s Disease

**Figure 5:**
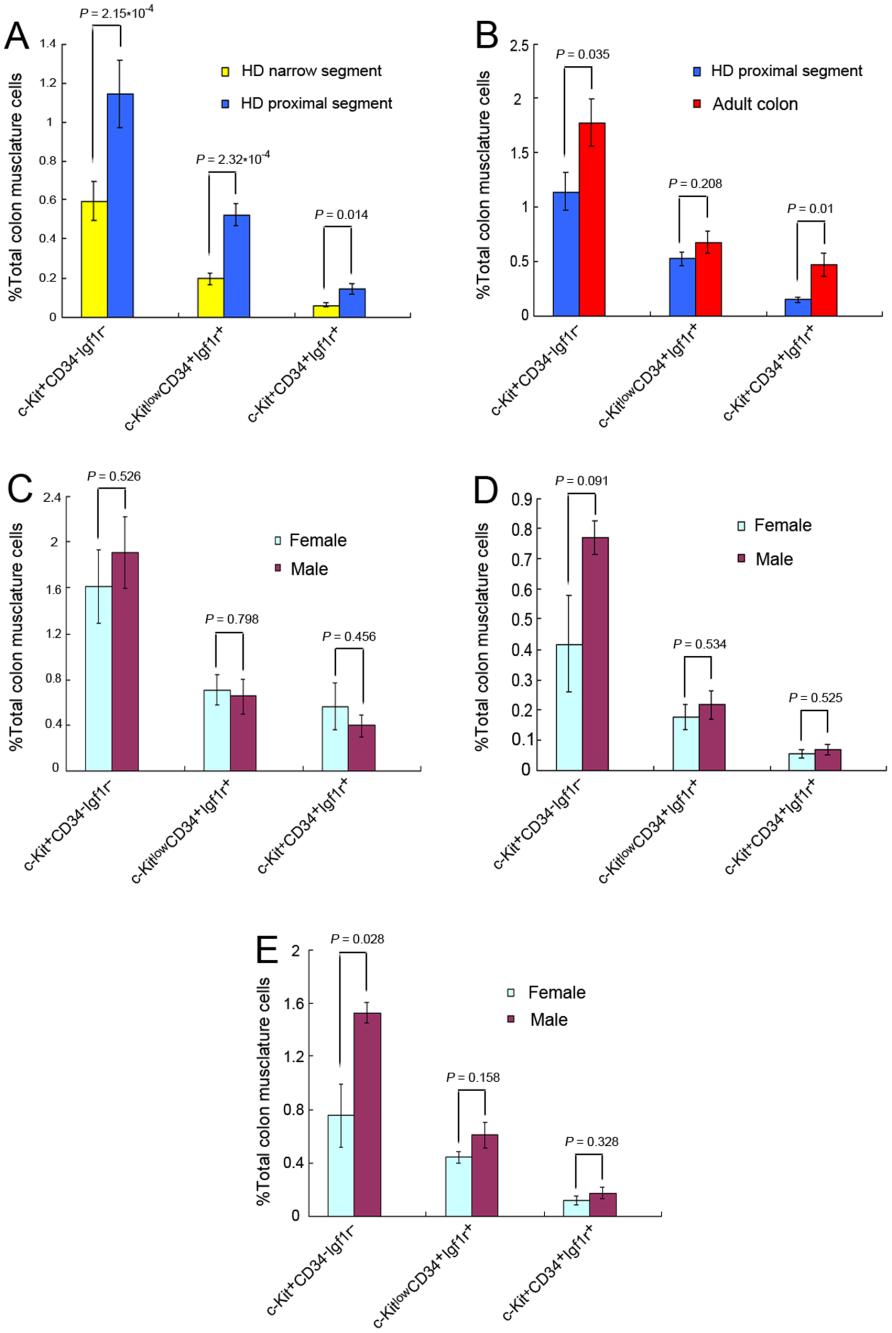
Statistical analysis of different proportions of ICC and progenitors in adult normal colon and narrow and proximal segments of HSCR colon. Numbers indicate total colon muscularis cell frequencies (%). (**A**) The proportions of mature, early and committed progenitors of ICC in the narrow segment of HSCR colon are all remarkably less than in the proximal segment. This is self-control by paired-sample *t* tests, (*n = *11). (**B**) In the proximal segment of the HSCR colon, the proportions of mature and committed progenitor of ICC differ from those of the normal adult colon (*n = *11); but the proportion of early ICC progenitor is similar to that of the normal adult colon (*n = *11). This is a comparison of different age groups by independent-sample *t* tests. (**C**) In adult normal colon, between the two genders, the proportions of mature, early and committed progenitors of ICC do not differ, as compared by self-controlled paired-sample *t* tests (female = 5, male = 6). (**D**) The narrow segment of the HSCR colon, which is the site of the lesion, shows no difference in the proportions of mature, early and committed progenitors of ICC between females (*n = *5) and males (*n = *6). They are compared by paired-sample *t* tests. (**E**) In the proximal segment of the HSCR colon, the mature ICC proportion in female colons is lower than in male colons, (female = 5, male* = *6). But the early and committed ICC progenitors show no difference. They are compared by paired-sample *t* tests. The detailed *P*-values were marked in the columns for each group. *P*<0.05 was used as cut-off for statistical significance.

In 11 cases of HSCR, the proportion of mature ICC in the proximal segment was 1.1435±0.173%. The proportion of mature ICC in the narrow segment was 0.5942±0.0993%. The data between narrow and proximal segments were compared as self-control by paired-sample *t* test (*n = *11, **Fig. 5A**). Regardless of gender, they had significant differences (*P = *2.15×10^−4^). The proportions of the early progenitor between proximal and narrow segments were significantly different (*P = *2.32×10^−4^), 0.5261±0.05813% and 0.1955±0.03021% respectively. The proportions of committed progenitor between proximal and narrow segments were also significantly different (*P = *0.014), 0.147±0.02684% and 0.0607±0.01034% respectively.

The data between different age groups for proximal segment of the HSCR colon and adult normal colon were compared by independent-sample *t* test (*n = *11, **Fig. 5B**). The percentage of mature ICC in the proximal segment of the HSCR colon was 1.1435±0.173%, which was fewer than in the adult normal colon, 1.7745±0.217% (*P = *0.035). The proportion of early progenitors in the proximal segment of HSCR colon was 0.6796±0.09892%, which was similar to the data in the adult normal colon (*P = *0.208). The proportion of the committed progenitors in the proximal segment of HSCR colon was 0.4719±0.10456%, which was fewer than in the adult normal colon (*P = *0.01).

We compared the proportions of mature ICC and its progenitors between the two genders by independent-sample *t* test. The proportions of mature ICC and its progenitors showed no discrepancy between adult male and female individuals (**Fig. 5C**). In the narrow segment of the HSCR colon, comparing males (*n = *6) and females (*n = *5), the proportions of mature ICC and its progenitors were not different (**Fig. 5D**). In the proximal segment of the HSCR colon, the proportion of mature ICC in females was fewer than in males (*P = *0.028). The early and committed progenitors of ICC in this part showed no difference between the two genders (**Fig. 5E**).

In summary, the proportions of mature, early and committed progenitors of ICC in the narrow segment of the HSCR colon were significantly reduced compared to the proximal segment and the adult normal colon. The detail *P*-values for the distribution of ICCs among the different groups were shown in the figure. **(** **Fig. 5A–E** **).**

### Ultrastructure Injury Changes in ICC of Hirschsprung’s Disease

**Figure 6:**
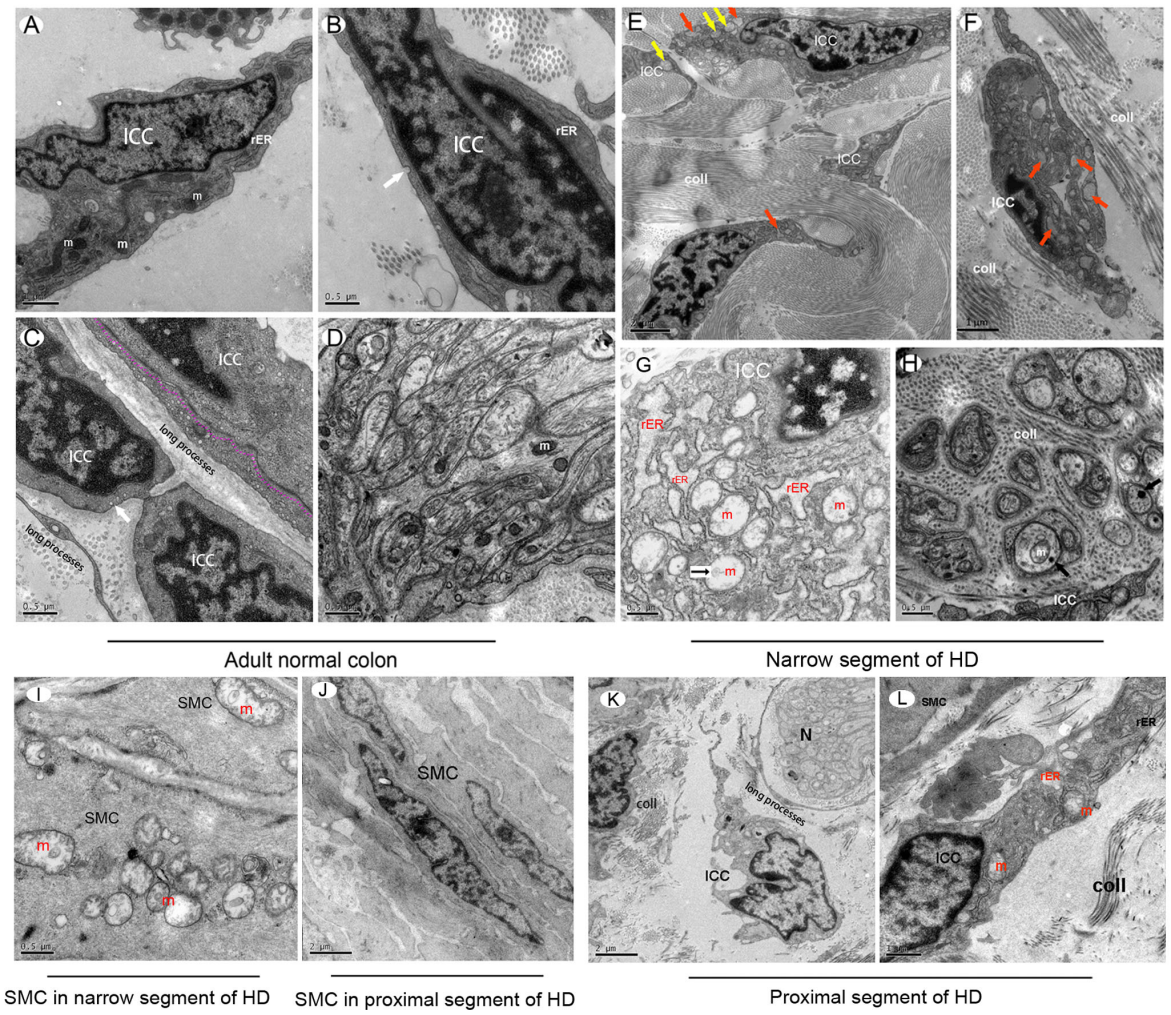
Ultrastructural changes in the narrow and proximal segment of the HSCR colon compared with normal adult colon. In normal human adult colon, typical ultrastructural features were observed by TEM (**A–D**). (**A**) Normal ICC had an oval nucleus with condensed heterochromatin distributed in the periphery, abundant mitochondria (m) and rough endoplasmic reticulum (rER). (**B**) *Caveolae* was located along the cell membrane (white arrow). (**C**) ICCs had long processes. Occasionally they were engaged in multicontact synapses with other ICC (pink dotted line). (**D**) In AP of human normal colon, there were normal mitochondria (m) inside of normal nerve bundles. (**E–G**) In the narrow segment of the HSCR colon, ICC showed evidence of severe injury. In almost all of the ICCs, the main features included swollen or vacuolated mitochondria, lack of mitochondrial cristae, dilatated and vesiculated rough endoplasmic reticulum (rER), and degranulated ribosomes. The *caveolae* on the ICCs plasma membrane were disappeared. Orange arrows in (**E**) and (**F**) indicate vesiculated rER. Yellow arrows in (**E**) indicate swollen or vacuolated mitochondria. (**E, F**) Injured ICCs were surrounded by large amounts of collagen fibrils (coll), ICC processes were hindered by these collagen fibrils from extending to form connections between cells. (**G**) High magnification displayed swollen or vacuolated mitochondria and vesiculated rER inside of ICC, which appeared as high density clumps inside of mitochondria (black small arrow). (**H**) Injured nerve bundles were surrounded by many collagen fibrils (coll). Inside of nerve bundles, swollen mitochondria and lysosomes were identified (black arrows). (**I**) Swollen or vacuolated mitochondria and dilatated rER inside of smooth muscle cells (SMC) in the narrow segment of the HSCR colon were identified as high density clumps present inside of some SMC mitochondria, which were similar to ICC. (**J**) Smooth muscle cells in the proximal segment of the HSCR colon were relatively normal. (**K, L**) In the proximal segment of the HSCR colon, there were long processes of ICC surrounding nerve bundles, not hindered by collagen fibrils (**K**). There were some swollen mitochondria and dilatated rER inside of ICC (orange font), at the same time some ICC presented with normal rER (black font). A few collagen fibrils (coll) presented beside ICC, but the amount was not greater than in the narrow segment of the HSCR colon.

Classic ICC ultrastructure can be found by TEM in the normal adult colon. Cytological ICC properties identified by TEM [35] include: the presence of numerous mitochondria, abundant intermediate filaments, the presence of surface *caveolae,* and partially (variably) developed basal lamina. ICC also have well-developed smooth and rough endoplasmic reticulum (rER), and gap junctions connecting them to smooth muscles cells (SMC) and other adjacent ICC. The important difference between SMC and ICC is that ICC lack thick myofilaments. In this study, we demonstrated that ICC in the normal adult colon are consistent with these criteria and possess normal organelle ultrastructure **(** **Fig. 6A–D** **)**.

In all narrow segment samples of HSCR colon, the ultrastructural injuries were widely observed in ICC cytoplasm and processes **(** **Fig. 6E–G** **)**. The main features included: i) swollen or vacuolated mitochondria, lack of mitochondrial cristae, occasional presence of lamellar bodies inside of cells, lack of normal mitochondria; ii) dilatated and vesiculated rER as well as degranulated ribosomes; iii) ICC surrounded by large amounts of collagen fibrils, ICC processes that are hindered from extending to form connections with SMC and adjacent ICC; iv) disappearance of the *caveolae* on the membrane surface; v) depletion or vacuolization of the cytoplasm and significant reduction in electron density of the perinuclear cytoplasm; vi) condensed and darkened cell nuclei. Nerve structure surrounded by collagen fibrils, and shows swollen mitochondria **(** **Fig. 6H** **)**. Lysosomes sometimes appear inside nerve fibers **(** **Fig. 6H** **).** SMC showed similar injury as ICC, including swollen mitochondria and dilatated and vesiculated rER **(** **Fig. 6I** **)**. These changes usually appeared under hypoxic or toxic conditions.

In the proximal segment of the HSCR colon, most organelles of ICC, SMC and enteric nerves appeared normal **(** **Fig. 6J–L** **)**. Occasionally, swollen mitochondria or dilatated or vesiculated rER were detected inside the ICC **(** **Fig. 6L** **),** but these changes were not more severe than in the narrow segment.

### Fate of Colon Muscularis Cells in Culture

**Figure 7:**
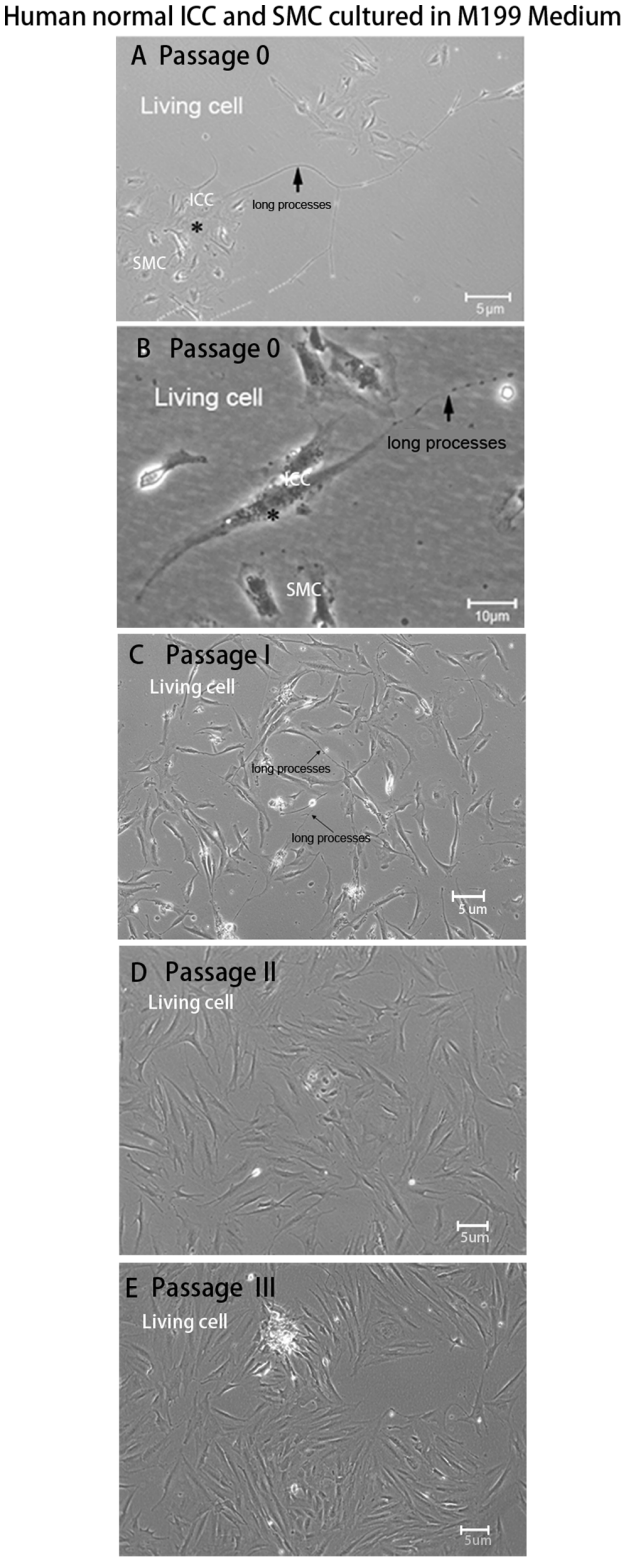
Morphological features of human normal ICC and smooth muscle cells cultured together. The total cells from adult normal colon muscularis including smooth muscle cells and ICC populations were cultured in M199 without SCF and IGF-I. These cells survived about 20 days. (**A, B**) Passage 0, the classic descriptions of ICC consistent with previous literature could be observed clearly under a light microscope. The ICC were stellate (**A**) or spindle (**B**) shaped, had a different number of ramified long cell processes, and tended to be in contact with smooth muscle cells in culture. (**C**) Passage 1, ICC with long processes presented in culture. (**D, E**) With increasing passage number, the primary cells gradually aged so that ICC could not be distinguished from muscle cells and fibroblasts.

The cells from adult normal colon muscularis, including SMC and ICC populations, were cultured in Medium 199 without SCF or IGF-I. These cells could survive about 20 days and 3–4 passages. With stimulation by human SCF and IGF-I, cells could survive more than 40 days (data not shown). Only in passage 0 and 1 could we observe ICC characteristics under light microscopy that were consistent with classic descriptions of ICC. The ICCs were spindle or stellate shaped, had a different number of ramified long cell processes, and tended to be in contact with SMC in culture **(** **Fig. 7A–C** **)**. With increasing passage number, the primary cells gradually aged so that ICC could not be distinguished from SMC and fibroblasts (**Fig. 7D, E**). However, the cells from the HSCR colon failed to survive under the same culture conditions.

## Discussion

Lőrincz *et al* first described the phenotype of ICC progenitor in the mouse gastrointestinal tract (33, 34). We designed this study to figure out whether such ICC progenitors exist in the human colon and determine their normal proportion. In addition we wanted to study what the changes of ICC and its progenitors occurred in the Congenital Hirschsprung’s disease. We identified the c-Kit^+^CD34^+^Igf1r^+^ cells existing inside the AP in adult normal colon by laser confocal localization and 3D reconstruction techniques. The cell population was almost non-exiting in narrow segment of HSCR.

It was difficult to distinguish c-kit^+^ and c-kit^low^ cells by immunofluorescence, so we used flow cytometry. We identified the c-kit^low^ cell populations by selecting cells between the c-Kit^+^ and c-Kit^−^ populations on flow cytometry, particularly on the abscissa of anti-c-Kit. However, the c-kit^low^ population was too small to observe and their numbers were significantly lower in cells derived from the narrow segment of HSCR colons. There was possible technical inadequacy in classification of c-kit expression levels. This study required greater quantities of total cells in order to detect adequate numbers of c-kit^low^ cells on flow cytometry.

Flow cytometry data not only showed the presence of ICC, but also demonstrated differences in their proportions between human adult normal colon and HSCR colon. Consistent with our speculation, in the narrow part of HSCR colon, the numbers of mature, early and committed progenitors of ICC were significantly reduced. There was not only a small amount of mature ICCs remain in the narrow part of the HSCR colon but also surrounded by thick collagen fibrils. We suggest that these cells are either not sufficient to carry out the intestinal pacemaker function or they can not launch electrical signals. This could be the reason why the HSCR patient has no physiological reflection in clinical rectal manometry.

ICCs are particularly susceptible to ischemia due to several reasons including the inability to visualize all ICC from formalin fixed, paraffin embedded tissue despite optimal antigen retrieval. So we were not able to use the tissue from frozen bodies such as body autopsy. In preliminary experiments, we tested the effect of different treatment time on the proportion of ICC. The whole procedure time included cell preparation and FACS. The procedure time was limited to 5 hours or 24 hours with or without SCF and IGFI. We found the data at 24 hours deviated from 5 hours very much.

Because it is difficult to collect fresh colon samples from healthy children, so we used adult colon samples to compare with the data of HSCR children samples in this study. Although we compared the data of HSCR children with those of adult group, we think the results presented here are very meaningful. Firstly, our data from the adult normal colon group support that ICC progenitors never disappear during aging. It was described that both in stomach and colon, the number of ICC bodies and volume significantly decreased with aging at a rate of 13% per decade of life [36]. But in that research the colonic tissues from patients age 36–92 years old not younger people even not children. The changing trend data of ICC and progenitors in colon from children to young adult is still unknown. In this research, the 11 cases performed same operation of one-Stage transanal Soave pullthrough surgery and one year follow-up. They had the similar result that restored self-defecation. Although their surgical age span was large from 3 to 36 month, there was no difference of the proportion of ICC and progenitors between younger and older 12 months groups, whatever in proximal or narrow segment. The mount of ICC and progenitors only associated with the position in HSCR. Further more, although the number of ICC in adult colon was affected by aging, the content of mature and progenitor ICC in the narrow segment of HSCR were far fewer than those in the normal adult colon. The average age of adult group was 63.53 years old in our study and the number of ICC was affected by aging. This result suggests that the proliferation and differentiation of ICC progenitors in the narrow segment of the HSCR colon may be damaged more significantly by pathogenic factors *in utero* than by physiological aging.

The numbers of mature and committed progenitor ICC in the proximal part of the HSCR colon are fewer than in adult normal colon; but the proportion of early progenitor ICC in this part of the HSCR is similar to adult colon. Because the narrow part of HSCR colon was removed during surgery, the proximal segment was pulled down to be the position of the intestinal tract reconstruction. All of the HSCR children in this study were able to regain normal bowel function gradually after surgery. We speculated that the regeneration of mature and progenitors of ICC improved so that their physiological pacemaker function recovered.

In addition, we found that there were no differences in the proportions of ICC and their progenitors between males and females whether in adult normal colon or in the narrow segment of HSCR colon. This means that in both genders, for HSCR patients, the narrow part of the colon suffered same damage. But in the proximal segment of the HSCR colon, the proportion of mature ICC in females was less than in males. However, their prognoses were the same, and this result may have been caused by small sample size. In the future, we can expand the number of HSCR colon samples to confirm this result.

Furthermore, TEM findings about cell ultrastructure confirmed our hypothesis that many important organelles appeared injured. The mitochondrion is a site of biological oxidation within the cell [37]. The citric acid cycle, respiratory electron transport chain and oxidative phosphorylation all happen within it. Mitochondria also have many other functions in addition to the production of ATP, such as transiently storing calcium, a contributing process for the cell’s homeostasis of calcium [38]. The concentrations of free calcium in the cell can regulate an array of reactions and is important for signal transduction in the cell. Therefore, as a power plant, mitochondria participate in cell differentiation, cell information transfer, apoptosis, and regulation of cell growth and cell cycle [39]. The rough endoplasmic reticulum (rER) is an important place of protein synthesis, including secreted protein, peptide hormones, growth factors, enzymes and integral membrane proteins. Cell surface antigens and receptors comprise a major category of membrane proteins. Lack of such membrane antigens and receptors prevents cells from accepting growth factors from the environment and carrying out intracellular physiological activities that cause cell survival, proliferation, and differentiation processes to be hindered. We found that in the narrow segment of the HSCR colon, ICC injury changes were widely visible. These changes suggest that energy metabolism and protein synthesis are decreased, defective or absent. Two vital organelles were injured, which could greatly affect normal biological function and cell survival. This may be the reason for the decreased number of ICC and its progenitors in the narrow segment of the HSCR colon. This reason can also explain why the cells from the HSCR colon can’t be cultured *in vitro*.

Another cell that may have similar ultrastructural features to ICC is the fibroblast, but fibroblasts do not have *caveolae* and rarely have smooth *cisternae,* intermediate filaments, or a partial basal lamina [35]. It is reported that Igf1r^+^/CD34^+^ ICC as progenitor cells are identified ultrastructurally as fibroblast-like ICC (FL-ICC) in Ws/Ws rat colon, because of losing *caveolae* on the membrane surface [40]. *Caveolae* is a calcium channel related to gut electrophysiological pacing function. Ws/Ws rats lose ICCs which have pacemaker function and have hardly any c-Kit^+^ reaction at the level of AP. Our result was consistent with this. In the narrow segment of the HSCR colon, *caveolae* were absent or lined the ICC cell membrane. At the same time, there were a lot of collagen fibers surrounding the ICC. We speculated ICC would transform into fibroblasts, and the physiological signal conduction between ICCs and nerves would be prevented. These observations combined with the loss of mitochondrial energy production, may be the cause for the loss of pacemaker function in ICC.

We attempted to detect the sorted ICC progenitors by TEM, but due to disease lesion, the number of sorted cells was significantly lower than the minimum requirement for TEM detection so we failed to generate an ultrastructural image. However, the ultrastructural injury changes were widely visible in the narrow segment of the HSCR colon, not only in ICC but also in the enteric nervous and smooth muscle cells. We suggest that the progenitors of ICC may also be injured by some pathogenic factors that exist in the narrow segment of the HSCR colon. Once the proliferation and differentiation capacities are compromised, the progenitors of ICC may not be able to regenerate the mature ICC network. The pathogenesis of HSCR is not completely clear yet. The majority view is that HSCR is a multifactor genetic disease, caused by the interaction of genetic and environmental factors. Analysis of the TEM results suggests that the pathogenic factors may involve fetus ischemia and hypoxia and intrauterine infection or toxin, which would lead to ICC and its progenitors undergoing abnormal development and survival.

## Conclusions

In summary, our results demonstrate the existence of ICC progenitors in normal adult and HSCR children colon, but their numbers are varied in relation to clinical status. We speculate that the fetus may be affected by inflammation, toxins, or hypoxic-ischemic injury stimuli in the maternal uterus or in the process of delivery so that ICC, ICC progenitors, and enteric nerves may suffer similar severe injuries. This kind of injury was more severe than that from physiological aging. Our next step is to explore the molecular biology process and mechanisms underlying these injuries. In the present study, we have established a method for purifying ICC progenitors from fresh colon tissue, which is very helpful for the further study of pathological molecular biology of HSCR. Additionally, establishment of *in vitro* culture systems to regulate the growth, proliferation and differentiation of purified normal ICC progenitors by some appropriate specific growth factors will help to restore the recession of gut function due to aging, diabetes, injury or congenital disorder.

## Supporting Information

Figure S1 **The sampling sites show on the colon of HSCR.** The arrows showed the narrow and proximal part of HSCR colon. The narrow part is the lesion site. The proximal part is the site for surgical reconstruction which was identified as well-developed ganglion by frozen section during the operation.
(TIF)

Figure S2 **Distinguishment of stained cells from the nonspecific immunoreactivity.** The total cell population (R1) was used to detect the expressing CD34^+^/Igf1r^+^ population. The location of double-positive CD34^+^/Igf1r^+^ cells was in R9 region in flow cytometry assay. The nonspecifically labeled cells located in R8, were very different from the population in R9 region in flow cytometry assay.
(TIF)

Figure S3 **Mature and progenitor ICC localization in human adult normal colon examined by laser confocal microscope and 3D reconstruction.** **(A–D)** At 200×magnifications, the c-Kit positive mature ICCs connected to each other formed extending chords and around AP. Inside of AP (white frame), some c-Kit positive cells also expressed CD34^+^/Igf1r^+^. **(E–H)** The immunofluorescences of ICC progenitors in the white frame were better visualized under 400×magnifications. ICC progenitors were c-Kit^+^CD34^+^Igf1r^+^, which were different from enteric nerve which also expressed Ifg1r. **(I, J)** Three-dimensional reconstruction of c-Kit^+^ and c-Kit^+^ CD34^+^/Igf1r^+^cells located in normal adult colon. Green, red and pink fluorescence represent c-Kit, CD34 and Igf1r, respectively.
(TIF)

File S1 **Tables S1–S4.** Table S1. HSCR Patients’ information and follow-up result. This table showed the information of HSCR group, anorectal manometry before surgery and the result of follow-up. The HSCR children restored their self-defecation after surgery without recurrence of constipation or/and bloating consider as “Good”. Table S2. Information of adult group. This table showed adult group information including: surgical age, sexual, sample collection site and intestinal function. Table S3. The proportions difference of cells in the proximal segment between younger and older 12 months groups. This table showed in the proximal segment of HSCR, there was no difference of the proportions of ICC, early and committed progenitors between younger and older 12 months groups. *P> 0.05* ranked as no difference. So the data from proximal of 11 cases were generalized in one group to analyze. Table S4. The proportions difference of cells in the narrow segment between younger and older 12 months groups. This table showed in the narrow segment of HSCR, there was no difference of the proportions of ICC, early and committed progenitors between younger and older 12 months groups. *P> 0.05* ranked as no difference. The degree of cells decrease was the same so the data from narrow segment of 11 cases were incorporated into one group to analyze.
(DOC)

Flowchart S1 **Sample collection and use of process.** In this research, 11 case of HSCR samples and 11 normal adult colons were collected. This flowchart showed how the samples were used for immunofluorescence, flow cytometry, culture *in vivo* and TEM. The method about the data analysis and comparison of cell frequencies by FACS were showed at the same time.
(DOC)
