# Supplementary figures and images for: Characterization of Interstitial Cajal Progenitors Cells and Their Changes in Hirschsprung’s Disease
Source: PLoS One. 2014 Jan 24;9(1):e86100. doi: 10.1371/journal.pone.0086100 (PMC3901676; doi:10.1371/journal.pone.0086100)

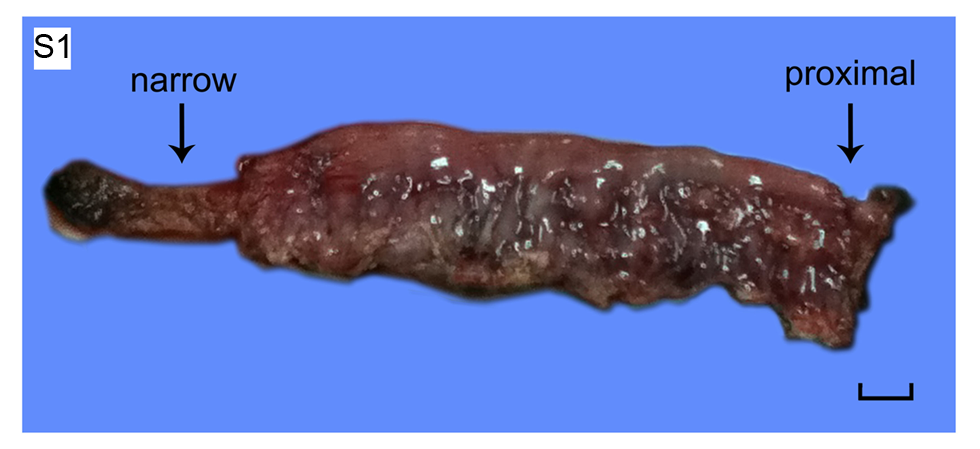

Supplement: Figure S1 — The sampling sites show on the colon of HSCR. The arrows showed the narrow and proximal part of HSCR colon. The narrow part is the lesion site. The proximal part is the site for surgical reconstruction which was identified as well-developed ganglion by frozen section during the operation. (TIF) [file pone.0086100.s001.tif]

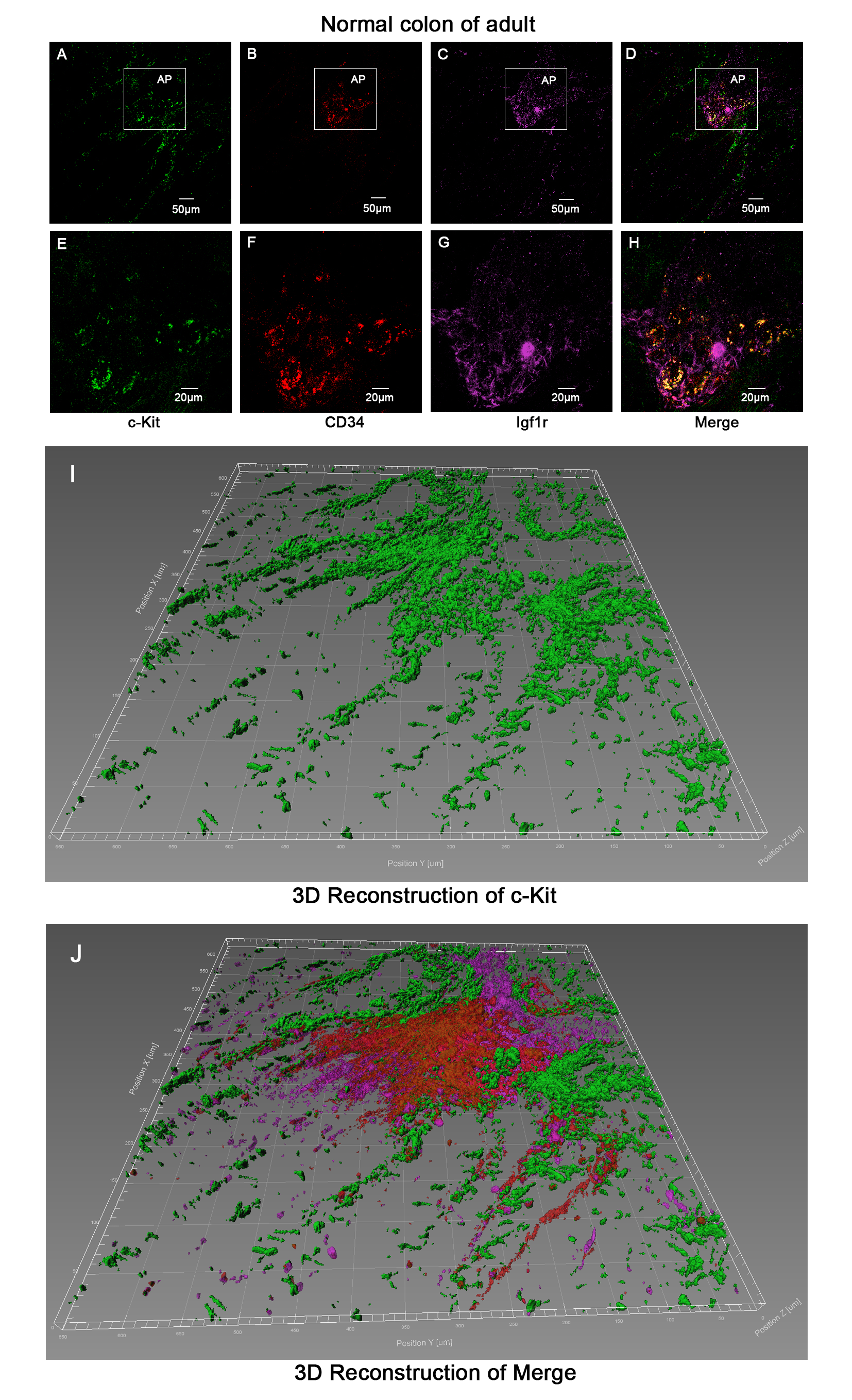

Supplement: Figure S2 — Distinguishment of stained cells from the nonspecific immunoreactivity. The total cell population (R1) was used to detect the expressing CD34+/Igf1r+ population. The location of double-positive CD34+/Igf1r+ cells was in R9 region in flow cytometry assay. The nonspecifically labeled cells located in R8, were very different from the population in R9 region in flow cytometry assay. (TIF) [file pone.0086100.s002.tif]

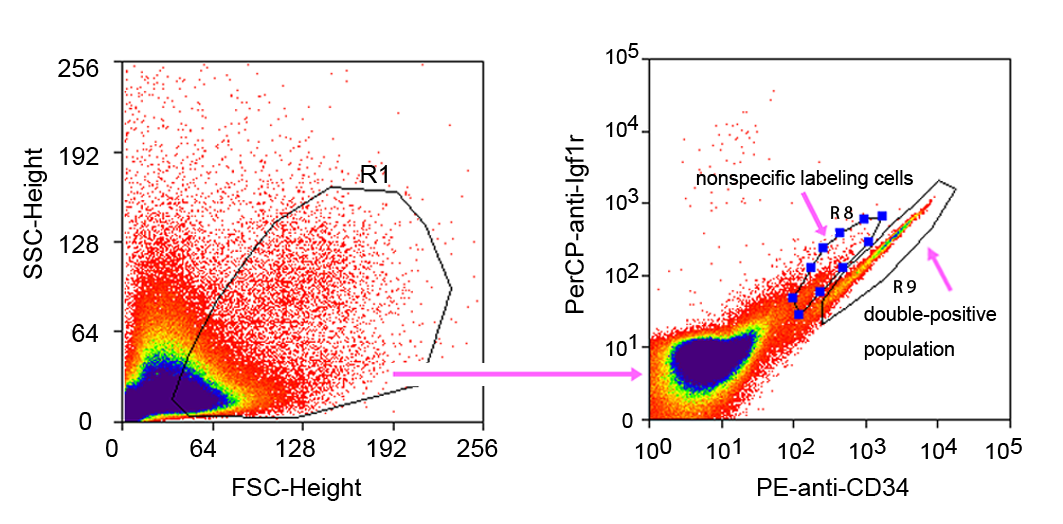

Supplement: Figure S3 — Mature and progenitor ICC localization in human adult normal colon examined by laser confocal microscope and 3D reconstruction. (A–D) At 200×magnifications, the c-Kit positive mature ICCs connected to each other formed extending chords and around AP. Inside of AP (white frame), some c-Kit positive cells also expressed CD34+/Igf1r+. (E–H) The immunofluorescences of ICC progenitors in the white frame were better visualized under 400×magnifications. ICC progenitors were c-Kit+CD34+Igf1r+, which were different from enteric nerve which also expressed Ifg1r. (I, J) Three-dimensional reconstruction of c-Kit+ and c-Kit+ CD34+/Igf1r+cells located in normal adult colon. Green, red and pink fluorescence represent c-Kit, CD34 and Igf1r, respectively. (TIF) [file pone.0086100.s003.tif]
