# Supplementary material for: Characterization of Interstitial Cajal Progenitors Cells and Their Changes in Hirschsprung’s Disease
Source: PLoS One. 2014 Jan 24;9(1):e86100. doi: 10.1371/journal.pone.0086100 (PMC3901676; doi:10.1371/journal.pone.0086100)
Supplement: File S1 — Tables S1–S4. Table S1. HSCR Patients’ information and follow-up result. This table showed the information of HSCR group, anorectal manometry before surgery and the result of follow-up. The HSCR children restored their self-defecation after surgery without recurrence of constipation or/and bloating consider as “Good”. Table S2. Information of adult group. This table showed adult group information including: surgical age, sexual, sample collection site and intestinal function. Table S3. The proportions difference of cells in the proximal segment between younger and older 12 months groups. This table showed in the proximal segment of HSCR, there was no difference of the proportions of ICC, early and committed progenitors between younger and older 12 months groups. P> 0.05 ranked as no difference. So the data from proximal of 11 cases were generalized in one group to analyze. Table S4. The proportions difference of cells in the narrow segment between younger and older 12 months groups. This table showed in the narrow segment of HSCR, there was no difference of the proportions of ICC, early and committed progenitors between younger and older 12 months groups. P> 0.05 ranked as no difference. The degree of cells decrease was the same so the data from narrow segment of 11 cases were incorporated into one group to analyze. (DOC) [file pone.0086100.s004.doc]

**Table S1**

| Patient No | Age  (month) | Sex | Sample region | Rectal resting pressure (mmhg) | High  pressure zone(mmhg) | rectal manometry  reflection | Bowel function  In Follow-up |
| --- | --- | --- | --- | --- | --- | --- | --- |
| 1 | 3 | female | rectosigmoid | 10.3 | 35.4 | None | Good |
| 2 | 4 | female | rectosigmoid | 9.8 | 45.8 | None | Good |
| 3 | 36 | female | rectosigmoid | 16.2 | 44 | None | Good |
| 4 | 7 | male | rectosigmoid | 9.1 | 40 | None | Good |
| 5 | 13 | female | rectosigmoid | 13.3 | 33 | None | Good |
| 6 | 3 | male | rectosigmoid | NA | NA | NA | Good |
| 7 | 4 | female | rectosigmoid | 9.3 | 38.22 | None | Good |
| 8 | 7 | male | rectosigmoid | 10.9 | 35.7 | None | Good |
| 9 | 21 | male | rectosigmoid | 7.6 | 46 | None | Good |
| 10 | 7 | male | rectosigmoid | NA | NA | NA | Good |
| 11 | 9 | male | rectosigmoid | 10.9 | 26 | None | Good |

**Table S2**

| Patient No | Age (year) | Sex | Sample region | Intestinal obstruction |
| --- | --- | --- | --- | --- |
| 12 | 52 | male | rectosigmoid | None |
| 13 | 39 | male | rectosigmoid | None |
| 14 | 72 | female | rectosigmoid | None |
| 15 | 56 | male | rectosigmoid | None |
| 16 | 68 | female | rectosigmoid | None |
| 17 | 53 | female | rectosigmoid | None |
| 18 | 66 | female | rectosigmoid | None |
| 19 | 81 | female | rectosigmoid | None |
| 20 | 69 | male | rectosigmoid | None |
| 21 | 49 | male | rectosigmoid | None |
| 22 | 94 | male | rectosigmoid | None |

**Table S3**

| Proximal segment | <12m |  |  | >12m |  |  |  |
| --- | --- | --- | --- | --- | --- | --- | --- |
|  | M (%) | SD | SEM | M (%) | SD | SEM | P-value |
| kit+CD34-IGF1R- | 1.1758 | 0.50356 | 0.17803 | 1.0573 | 0.67014 | 0.3869 | 0.755 |
| kitlowCD34+IGF1R+ | 0.5438 | 0.19625 | 0.06938 | 0.479 | 0.11274 | 0.06509 | 0.61 |
| kit+CD34+IGF1R+ | 0.1568 | 0.09351 | 0.03306 | 0.121 | 0.02022 | 0.01168 | 5.41E-01 |

**Table S4**

| Narrow segment | <12m |  |  | >12m |  |  |  |
| --- | --- | --- | --- | --- | --- | --- | --- |
|  | M (%) | SD | SEM | M (%) | SD | SEM | P-value |
| kit+CD34-IGF1R- | 0.6215 | 0.30193 | 0.10675 | 0.5213 | 0.33726 | 0.19472 | 0.645 |
| kitlowCD34+IGF1R+ | 0.1643 | 0.08068 | 0.02852 | 0.2787 | 0.06334 | 0.03657 | 0.056 |
| kit+CD34+IGF1R+ | 0.0561 | 0.03385 | 0.01197 | 0.073 | 0.02211 | 0.01277 | 4.50E-01 |
