# Supplementary material for: Characterization of Interstitial Cajal Progenitors Cells and Their Changes in Hirschsprung’s Disease
Source: PLoS One. 2014 Jan 24;9(1):e86100. doi: 10.1371/journal.pone.0086100 (PMC3901676; doi:10.1371/journal.pone.0086100)
Supplement: Flowchart S1 — Sample collection and use of process. In this research, 11 case of HSCR samples and 11 normal adult colons were collected. This flowchart showed how the samples were used for immunofluorescence, flow cytometry, culture in vivo and TEM. The method about the data analysis and comparison of cell frequencies by FACS were showed at the same time. (DOC) [file pone.0086100.s005.doc]

**Flowchart S1**

11 samples of HSCR

11 samples of adult normal colon

laser-confocal (n = 5)

Living cells culture *in vivo* (n = 3)

TEM

(n = 3)

FACS

(n = 11)

Narrow segment of HSCR (n = 11)

Proximal segment of HSCR (n = 11)

Data analysis of cell frequencies

c-Kit^+^CD34^-^Igf1r^-^

**c-**Kit^low^CD34^+^Igf1r^+^

**c-**Kit^+^CD34^+^Igf1r^+^

Narrow *vs.* proximal part

Proximal part *vs.* normal adult colon

Female *vs.* male in narrow part

Female *vs.* male in proximal part

Female *vs.* male in normal adult colon
